# *In vitro* and *in vivo* study of additive manufactured porous Ti6Al4V scaffolds for repairing bone defects

**DOI:** 10.1038/srep34072

**Published:** 2016-09-26

**Authors:** Guoyuan Li, Lei Wang, Wei Pan, Fei Yang, Wenbo Jiang, Xianbo Wu, Xiangdong Kong, Kerong Dai, Yongqiang Hao

**Affiliations:** 1 Department of Orthopaedics, Shanghai Ninth People’s Hospital, Shanghai JiaoTong University School of Medicine, 639 Zhizaoju Road, Shanghai 200011, People’s Republic of China; 2 Shanghai Key Laboratory of Orthopaedic Implant, Shanghai Ninth People’s Hospital, Shanghai Jiaotong University School of Medicine, 639 Zhizaoju Road, Shanghai 200011, People’s Republic of China; 3 Research and Development Center of Medical Implant Engineering Technology, Engineering Research Center of Digital Medical and Clinical Translation Ministry of Education, 1954 Huashan Road, Shanghai 200011, People’s Republic of China; 4 Research and Development Department, Thytec Shanghai Co.,Ltd, 320 Xingda Road, Shanghai 201100, People’s Republic of China

## Abstract

Metallic implants with a low effective modulus can provide early load-bearing and reduce stress shielding, which is favorable for increasing *in vivo* life-span. In this research, porous Ti6Al4V scaffolds with three pore sizes (300~400, 400~500, and 500~700 μm) were manufactured by Electron Beam Melting, with an elastic modulus range of 3.7 to 1.7 GPa. Cytocompatibility *in vitro* and osseointegration ability *in vivo* of scaffolds were assessed. hBMSCs numbers increased on all porous scaffolds over time. The group with intended pore sizes of 300 to 400 μm was significantly higher than that of the other two porous scaffolds at days 5 and 7. This group also had higher ALP activity at day 7 in osteogenic differentiation experiment. The scaffold with pore size of 300 to 400 μm was implanted into a 30-mm segmental defect of goat metatarsus. *In vivo* evaluations indicated that the depth of bone ingrowth increased over time and no implant dislocation occurred during the experiment. Based on its better cytocompatibility and favorable bone ingrowth, the present data showed the capability of the additive manufactured porous Ti6Al4V scaffold with an intended pore size of 300 to 400 μm for large segmental bone defects.

Bone provides a living system with necessary rigidity. Accidents, infections, bone tumors and other diseases make bone repair and regeneration a substantial challenge in orthopedics. Major bone reconstruction methods usually use autografts or allografts with good biocompatibility to enhance bone union; however, these bone grafts suffer from numerous limitations, making synthetic alternatives a fascinating option^1^,^2^. Calcium phosphates act as synthetic bone graft substitutes because they are osteoconductive and exhibit the possibility of biodegradation; however, they also have the risk of implant failure due to insufficient mechanical properties along with scaffold degradation^3^. Titanium and titanium alloys are widely used for manufacturing implants in orthopedic and dental clinics because of their good biocompatibility, corrosion resistance and osseointegration properties^4^,^5^,^6^.

Implants embedded in the body should provide sufficient mechanical support to facilitate bone regeneration. Meanwhile, mechanical properties, such as stiffness, must be consistent with the surrounding bone tissue to acquire longevity by averting so-called stress shielding, which has an influence in bone remodeling and healing process^7^. In addition, the properties of bone tissue are influenced by human age, physical activity, nutrition and other related sickness, making it difficult to design and manufacture implants with similar mechanical and biological properties for bone repair and regeneration, especially in large segmental bone defects. There are two types of bone in human skeleton, namely, cortical and trabecular bones, with an elastic modulus range from 0.5 GPa to 20 GPa, which is much lower than that of dense titanium alloys (about 114 GPa)^8^. This modulus mismatch has been deemed as one of the primary reasons for stress shielding of bone^9^,^10^. To mimic bone properties, implants that have a porous structure may be an effective approach to eliminate this mismatch while maintaining the material composition^11^. Furthermore, the porous structure allows for vascularization and bone ingrowth, which could increase the interfacial bond between implant surface and host tissue, that is, the porous morphology offers stable long term fixation via biological anchorage of the implant. Last, porous metallic materials can initially provide mechanical stability after being embedded, which cannot be ignored when repairing large segmental bone defects within load-bearing areas. In fact, porous titanium and titanium alloys have been shown to possess excellent mechanical properties, enabling their use in permanent orthopedic implants under load-bearing conditions^12^.

For these reasons, there is a demand for fabrication procedures for porous metallic materials that could adjust the pore size and distribution, porosity and mechanical properties of implants for medical applications. Traditional fabrication procedures include powder sintering, plasma spray coating and foam fabricating^13^,^14^, while implants manufactured with these procedures are brittle and can easily crack at low stress^13^,^14^. Other techniques using foam agents suffer from limitations, such as impurity, contamination and predetermined part geometries. In addition, the most important issue is that the porosity, pore size and distribution, overall shape and volume fraction of implants are difficult to control; all of these characteristics have a primary influence on their biological and mechanical properties. Fortunately, with the development of additive manufacturing (AM), also commonly known as 3D printing, implants with different geometric shapes can be acquired by altering the computer aided design (CAD) model using computed tomography or magnetic resonance imaging data^15^. Among the many additive manufacturing technologies, recently, electron beam melting (EBM) has been widely used due to easily control the mechanical properties and precise adaptation to the region of implantation while eliminating secondary processing^8^. EBM is a solid freeform fabrication process that uses electron beam energy as power, overlaying materials layer by layer based on CAD data. During the fabrication process, the focused electron beam is rastered over each successive layer of powder, which is gravity-fed from powder cassettes and raked into successive layers. This technique allows us to fabricate complex products with freeform shapes and porous structures by controlling the pore size and porosity to meet a variety of different applications requirements. Recently, this procedure has been used to fabricate porous implants that have been reported to have favorable biological, physical and mechanical properties^16^,^17^.

To date, many publications have indicated that micro-porosity is vital for biomaterials to achieve promising osteo-induction^18^,^19^. A porous structure with interconnected pores is conducive to the exchange of nutrients and vascularization, thereby fulfilling bone ingrowth to achieve long-term fixation by biological anchorage^20^. In addition, pore shape and distribution is important for osteointegration, which also relies on the material surface topography^21^,^22^. Both the pore size and porosity play critical roles in bone ingrowth. Previous research has shown that the optimal pore size for bone ingrowth is in the range of 100 to 500 μm^23^,^24^,^25^. Another study has indicated that porous morphology with pore size larger than 150 μm provided that there is a good environment for the ingrowth of natural bone^26^. Alternatively, pore sizes greater than 300 μm were recommended for enhancing new bone formation and the formation of capillaries^27^. Despite numerous similar reports, to date, the scientific community has not reached a consensus with respect to the optimum porosity and pore size for bone ingrowth.

The purpose of this study was to explore the effect of pore size and porosity on the mechanical and biological characteristics of additive manufactured porous Ti6Al4V scaffolds. Therefore, porous scaffolds with three pore sizes (300~400, 400~500, and 500~700 μm) and the corresponding porosities (33.8%, 50.9%, and 61.3%) were manufactured by EBM. A scanning electron microscope (SEM) was used to observe the porous structure, and a compression test was performed to characterize these porous scaffolds. The effect of pore size on cytocompatibility was evaluated *in vitro* with human bone mesenchymal stem cells (hBMSCs). Moreover, we conducted *in vivo* experiments to evaluate the biological performance of mechanical-adapted porous Ti6Al4V alloy scaffolds in large segmental metatarsal bone defects of goats at each time point.

## Methods

### Implant design and manufacturing

**Figure 1:**
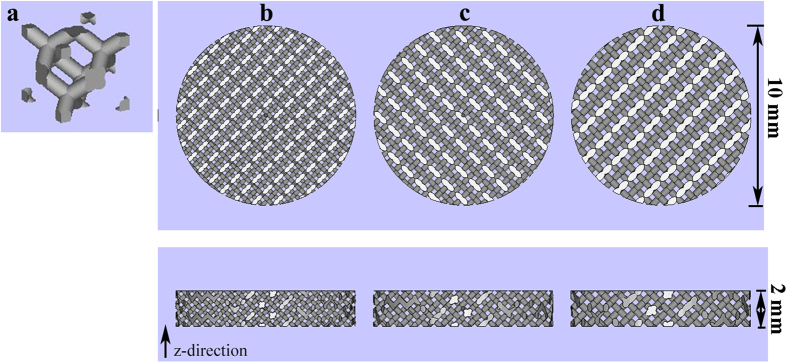
Computer aid design of porous Ti6Al4V alloy scaffolds with different pore size used *in vitro*. (**a**) A single unit of diamond-shaped lattice; (**b**) pore size of 300~400 μm; (**c**) pore size of 400~500 μm; (**d**) pore size of 500~700 μm.

**Figure 2:**
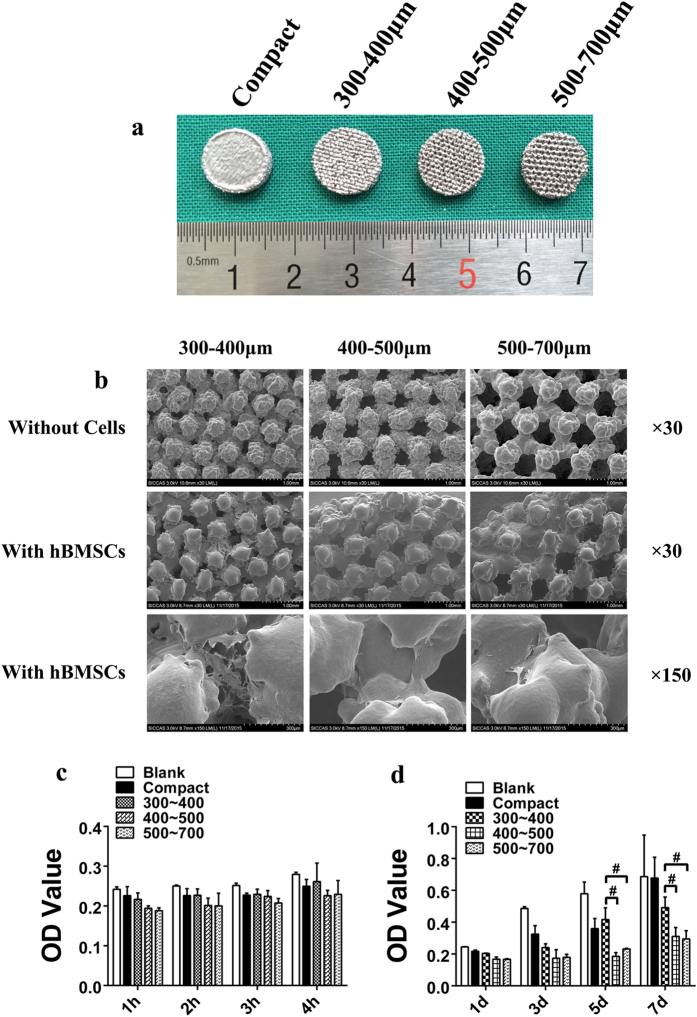
Scaffold appearance and cell adhesion, growth and morphology on the tested scaffolds. (**a**) Scaffolds used in the *in vitro* experiment with a height and diameter of 2 and 10 mm, respectively. (**b**) SEM micrographs of porous Ti6Al4V scaffolds and cell morphology on porous scaffolds after being cultured for 7 days. The cells were mainly located between the gaps of the struts, flattened and spread well with numerous filopodia extensions, and the cell number in the group of intended pore size of 300 to 400 μm was higher than that of the other two groups. (**c**) Cell adhesion on the tested scaffolds. A higher OD value indicates that more cells adhered on the scaffolds. (**d**) Cell growth on the tested scaffolds. A higher OD value indicates that more cells grew or remained “alive” on the scaffolds (^#^p < 0.05).

**Figure 3:**
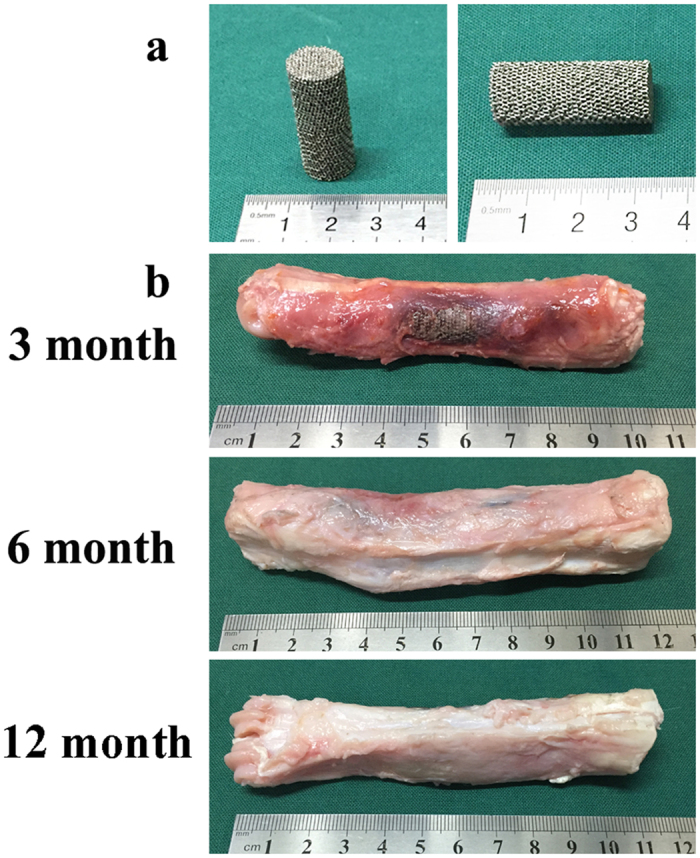
Scaffolds used in the *in vivo* experiment with a height and diameter of 30 and 10 mm (**a**) gross specimens at different time points (3 months, 6 months and 12 months) (**b**).

A standard Arcam Ti6Al4V extra low interstitial (ELI) powder (particle size of 45 ~ 100 μm) with a nominal composition of 6.04% Al, 4.05% V, 0.013% C, 0.07% Fe, 0.13% O, less than 0.005% N and H, and the balance Ti (in weight per cent) was used as the starting material^28^. Scaffolds were fabricated by an EBM system (Arcam A1, Arcam AB, Mölndal, Sweden) based on the CAD (software: Magics, version 18.03, Belgium) data with various designs (Fig. 1). And the EBM system schematic has been described in detail previously^29^. Three types of porous Ti6Al4V alloy scaffolds were designed with intended pore sizes of 300~400, 400~500, and 500~700 μm. In addition, another group of compact Ti6Al4V alloy scaffolds was designed as the control. All four groups of Ti6Al4V alloy scaffolds exhibited a height and diameter of 2 and 10 mm for *in vitro* experiments, respectively (Fig. 2a). The layer thickness of the build process was 5 μm and porous scaffolds were manufactured with a same geometrical structure of diamond-shaped lattice. The designed strut size of three porous scaffolds (300~400, 400~500, and 500~700 μm) was 0.2 mm, 0.3 mm, and 0.3 mm respectively. All scaffolds were built in the z-direction of the cylinder and were manufactured with a beam power of 300 W at an average build temperature of 680 °C. Electron beam scanning speed was about 400 mm/s and scaffolds were protected in a helium atmosphere before being taken out. The fixation plate and screws used in animal study was manufactured with titanium alloys. Plate size was 72 × 9 mm or 88 × 9 mm with a thickness of 2 mm. Screws were 16 mm or 14 mm in length, with a shaft outer diameter of 2.5 mm and shaft inner diameter of 1.5 mm. Porous scaffolds were used for compression tests and *in vivo* experiments and exhibited a height and diameter of 30 and 10 mm, respectively (Fig. 3a). The microstructure, pore size and pore morphology of the fabricated porous Ti6Al4V alloy scaffolds were visualized using SEM (SU8220, HITACHI, Japan; imaging mode of low magnification; accelerating voltage of 3.0 kV; working distance of 8.7 mm or 10.6 mm) and analyzed by Image J 1.48 (National Institutes of Health; USA). The porosity of the samples was determined using the gravimetric method^30^.

Each of the scaffolds was sonicated for 30 min in ethyl alcohol (75%) and distilled water after the fabrication process to remove impurities and loose titanium particles. All of the scaffolds were sterilized by autoclaving at 121 °C for 20 min.

### Mechanical evaluations

Cylindrical specimens that had a diameter of 10 mm and a height of 30 mm were used to evaluate the elastic modulus and compressive strength of porous Ti6Al4V alloy scaffolds of different pore sizes (300~400, 400~500, and 500~700 μm) and porosities (33.8%, 50.9%, and 61.3%). The compression test was conducted using a computer controlled universal testing machine (Instron-5569, INSTRON, USA) at a crosshead speed of 1 mm/min. The elastic modulus of porous Ti6Al4V scaffolds was determined from the linear region of the stress–strain curve. Three specimens in each group were measured to obtain the average value of the mechanical parameters.

### *In vitro* cell culture

Human bone mesenchymal stem cells (hBMSCs) were isolated and expanded with the method previously reported^31^. All studies were carried out in accordance with the policies of, and with approval from, the Ninth People’s Hospital of Shanghai Jiao Tong University. The donor (male, 32y) was healthy without inherited illnesses, metabolic disease, or other diseases that may affect the results. Briefly, bone marrow from donor was taken after written informed consent using guidelines approved by the Ethical Committee on the Use of Human Subjects at the Shanghai Jiao Tong University. The cells were cultured in α-MEM (GIBCO, Grand Island, NY, USA) supplemented with 10% FBS (Hyclone, Tauranga, New Zealand) and antibiotics (streptomycin 100 lg/ml, penicillin 100 U/ml; Hyclone, Logan, UT, USA) at 37 °C in a moist atmosphere with 5% CO_2_; every 2 days, the growth medium was changed. After 96 h, the non-adherent cells were removed and the adherent cells were washed twice with a-MEM. hBMSCs from passages 3~5 were detached by 0.25% trypsin, re-suspended in fresh culture medium, and used for the experiments described below.

### Viability and morphology of hBMSCs

Disc specimens that had a diameter of 10 mm and a height of 2 mm were used to test the cell adhesion, cell proliferation and cell morphology for different pore sizes and porosities. The cell morphology was visualized using a scanning electron microscope. The experiments were performed in triplicate, and three specimens were used in each experiment in every group.

hBMSCs were seeded at 2 × 10^4^ cells/ml on the surface of scaffolds placed in 24-well plates. The cells were incubated in growth medium at 37 °C in 5% CO_2_ for 1 h, 2 h, 3 h, and 4 h for the cell adhesion assay and 1 d, 3 d, 5 d, and 7 d for the cell proliferation assay, and the growth medium was changed every 3 days. Before evaluation, all scaffolds were moved into another new 24-well plate. The Cell Counting Kit-8 (CCK-8) (Dojindo Molecular Technology, Japan) was used to evaluate the viable cell numbers after 2.5 h of incubation after being added to each well. The optical density (OD) was measured using an ELX800 absorbance microplate reader (Bio-Tek, USA) at 450 nm.

hBMSCs were seeded at 10 × 10^4^ cells/ml on the surface of scaffolds plated in 24-well plates and then incubated for 7 days at 37 °C in 5% CO_2_ in growth media that was changed every 3 days. After carefully removing the growth medium, the scaffolds were washed with a phosphate buffer solution (PBS) solution 3 times, fixed in a 2.5% glutaraldehyde solution over night at 4 °C, and then dehydrated in a graded series of ethanol solutions (50%, 60%, 70%, 80%, 90%, and 100%) for 10 min, followed by drying in a 37 °C dryer. Last, all disc samples were coated with gold before examination with SEM.

### Osteogenic differentiation evaluation

hBMSCs were seeded at 10 × 10^4^ cells/ml on scaffolds in a 24-well plate at 37 °C in 5% CO_2_ for 7 and 21 days. After 3 days, when the cells completely attached to the scaffolds, the growth medium was discarded and replaced with osteogenic differentiation medium (HUXMA-90021, Cyagen, USA) that was then changed every 3 days. The alkaline phosphatase (ALP) activity is an early marker for osteoblast differentiation. The cells cultured in osteogenic differentiation medium for 7 days were used to test the ALP activity using the ALP Activity Assay Kit (P0321, Beyotime, China), following the manufacturer’s instructions. After carefully removing the growth medium, the scaffolds were washed with a PBS solution 3 times. Next, samples were moved into a new 24-well plate, and then, the cells were lysed with 0.5 ml of a 0.2% Triton X-100 solution. The substrates and p-nitrophenol were subsequently added and incubated for 10 min at 37 °C. Finally, the ALP activity was determined at a wavelength of 405 nm.

At the end of the culture period of 21 days, the cells in the 24-well plate were washed 2 times with PBS and then fixed by 4% paraformaldehyde for 30 minutes. After staining with alizarin red for mineralized nodules, the scaffolds were rinsed 3 times in PBS. Thereafter, mineralized nodules were dissolved with 10% cetylpyridinium chloride (C9002-25G, Sigma, USA) for semi-quantitative analysis by examining absorbance at 562 nm. The experiments were performed in triplicate, and three specimens were used in each experiment in every group.

### Animal studies

The study was performed in compliance with the Chinese laws on animal experimentation and was approved by the Ethical Committee of Shanghai Jiao Tong University (Reference number: SYXK (Hu) 2012-0007 and SCXK (Hu) 2009-0018). Fifteen mature goats, non-pregnant and non-lactating, with a 45 ± 5 kg average weight, were acquired from an authorized experimental animal breeding company and were then acclimated for at least 4 weeks before surgery. Animals were randomly divided into three group for three different time periods (3 months, 6 months and 12 months postoperatively). The used implants were cylindrical scaffolds having intended pore size of 300 to 400 μm, diameter of 10 mm and a height of 30 mm.

The surgical procedure was performed via general intravenous anesthesia. Propofol (1%, 20 ml, Libang, China) was used to initialize and maintain the anesthesia. The right rear leg of the goats was shaved in the region of the metatarsus and then disinfected with ethyl alcohol (75%). After exposure by a lateral skin incision and blunt dissection, a bone defect was made using a fret saw along the longitudinal orientation under cooling with saline. The implants were press-fit inserted at their designated positions. To ensure the implant did not slip out and remained temporarily fixed, a plate held with six screws was placed beside the implant. The subcutaneous tissue and skin were then sutured. Metamizole (30 g) was intramuscularly delivered for five days postoperatively. The surgical area was stabilized with a cast for 1 month, and goats were restricted from moving with immediate loadbearing for 10 days.

At 3 months, 6 months and 12 months after implantation, the goats were killed with a lethal dose of sodium thiopental (80 mg per kg BW) via intravenous injection. Before euthanasia, gait videos were obtained. After sacrifice, the specimens of the metatarsus were collected for histological and histomorphometric analysis as well as radiography tests.

### X rays evaluation

To monitor the progress of osteointegration and bone healing, plain radiographs and computed tomography (CT) scans were performed after the animals were killed at each time point.

### Histological evaluation

All specimens were fixed in 10% paraformaldehyde for 7 days and then dehydrated in a graded alcohol series (70%, 80%, 90%, 99%, 100%, and 100%) for 3 days at room temperature. Then, specimens were embedded in methylmethacrylate (M55909, Sigma, USA) until solidification. The embedded sections were fabricated using a cutting-grinding system (Buehler 11-1280-250, USA) perpendicular to the axis of the implant and were then reduced to 80 μm. Along the longitudinal orientation of the implants, sections were obtained at three positions (both ends and the middle) to measure the depth of tissue ingrowth. After polishing, the sections were stained with Stevenel’s blue and Van Gieson’s picrofuchsin. The images were obtained using a Nikon SMZ 1500 stereoscopic zoom microscope (Nikon Instruments Inc.; Melville, NY). By the way, samples embedded in methylmethacrylate was also evaluated with SEM (SU8220, HITACHI, Japan; imaging mode of backscattered electron; accelerating voltage of 10.0 kV; working distance of 13.5 mm).

### Histomorphometric analysis

To perform quantitative analysis of the bone ingrowth area at three positions at different time periods, the images of the stained sections were measured via a computer using Image Pro-Plus 5.0 (Media Cybernetics; USA). The bone ingrowth area was defined as the bone area in the porous area available for bone ingrowth. Three different fields were examined for each section at the same magnification.

### Statistics

Data are expressed as the means ± standard deviation (SD). The data were analyzed with analysis of variance (ANOVA), followed by Fisher’s least-significant difference (LSD) method for pair-wise multiple comparisons. Statistical analysis was performed using SPSS 19.0. The differences were considered significant for p < 0.05.

## Results

### Implant characterization

The surface morphology of Ti6Al4V alloy scaffolds observed using SEM were presented with a three dimensional structure (Fig. 2b). The results showed that the porous scaffolds samples had a gradient porosity and a pore size corresponding with the intended pore size (Table 1).

### Mechanical evaluations

The compressive strength decreased from 115.2 MPa to 33.1 MPa, and the average elastic modulus decreased from 3.7 GPa to 1.7 GPa with increasing pore size. The specific data are indicated in Table 1. Overall, the elastic modulus of all three porous groups was less than the value of cortical bone, which can provide a promising mechanical stimulus on the surrounding bone tissue.

### *In vitro* cell adhesion, proliferation, morphology and osteogenic differentiation evaluations

Cell adhesion on all groups can be observed at each time point (Fig. 2b). The OD value increased in all groups with time (Fig. 2c). The cell adhesion was found to be positively correlated with time. However, there were no significant differences in the three porous groups. Cell migration ability was not visibly influenced by pore size in a short period of time.

The hBMSCs grew well, and the cell numbers increased in all samples with time (Fig. 2d). Thus, we could infer that porous Ti6Al4V alloy scaffolds fabricated by EBM exhibited promising cytocompatibility. Statistical analysis showed that cell viability on porous samples had no significant difference at days 1 and 3; however, the group of intended pore size of 300 to 400 μm was significantly higher than the other two porous scaffolds at days 5 and 7.

Figure 2b shows the SEM morphologies of hBMSCs on the scaffolds after 7 days of culturing. The cells could be observed on all scaffolds. The cells were mainly located between the gaps of struts. The cell morphology was flattened and spread well, with numerous filopodia extensions. Through general observation, the cell numbers in the group of intended pore size of 300 to 400 μm were apparently higher than those of the other two porous samples. Furthermore, cells on this porous scaffold became confluent and started to cover the pores, possibly due to the pore size being suitable for cell direct spanning. In the other two porous groups, the cell distribution was not uniform, which was adverse for cell growth and extracellular matrix formation.

**Figure 4:**
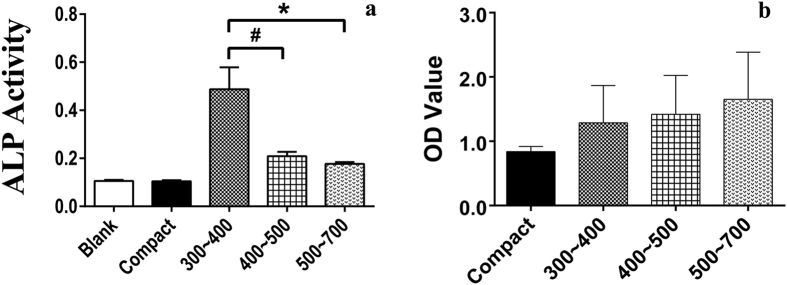
Cell osteogenic differentiation *in vitro*. ALP activity at day 7 (**a**) and semi-quantitative analysis of calcium nodule at day 21 (**b**) on porous Ti6Al4V scaffolds and the control samples. A high OD value indicates that much more calcium nodule formed on the scaffolds (#p < 0.05, *p < 0.01).

At day 7, ALP activity was detected in all groups, and the porous scaffolds were higher than the compact plate. The group of intended pore size of 300 to 400 μm was significantly higher than the other two porous scaffolds (Fig. 4a). The semi-quantitative analysis results of mineralized nodules are presented in Fig. 4b for day 21. Mineralized nodules were detected on all four scaffolds. It was obvious that the porous groups were superior to the compact Ti6Al4V alloy plate, with the osteogenic differentiation result being positively correlated with pore size, based on the trend in the figure. However, in fact, there were no significant differences in the three porous groups.

### *In vivo* gross inspections, X rays, histological and histomorphometric evaluations

All animals recovered well from anesthesia and the operative procedure. No implant dislocation and incision infection occurred during the experiment. Adverse reactions were not observed around the implants. Porous Ti6Al4V alloy scaffolds were stable and united with host bone at each of the sacrifice time points (Fig. 3b).

**Figure 5:**
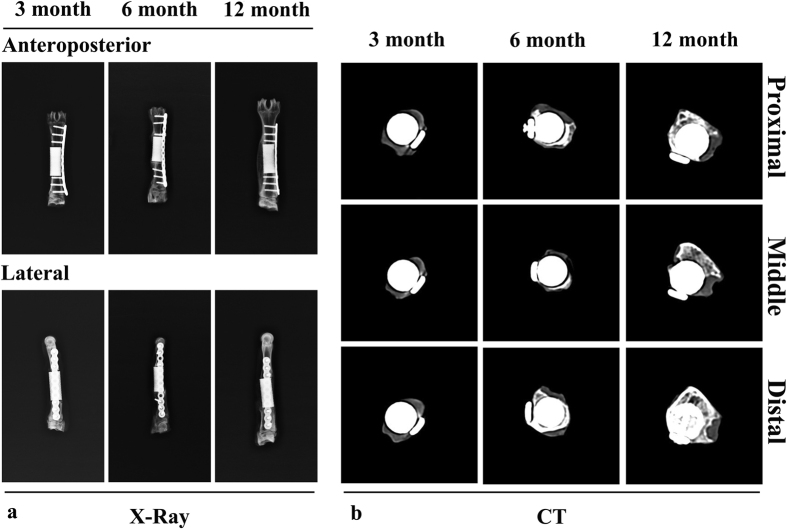
Plain radiographs (anterior-posterior and lateral) (**a**) and CT scans of specimens (proximal, middle and distal positions) (**b**) at different time points (3 months, 6 months and 12 months). New bone formation appeared on the lateral side opposite to the plate, and continuous mature bone was observed around the scaffolds at 1 year.

Plain radiographs and CT revealed that new bone growth occurred in all animals and that none of the implants were displaced (Fig. 5). At 3 months, callus could be observed outside of the implants in CT images. At 6 months, new bone was observed at both ends of the implant in plain radiographs. In addition, remodelled bone tissue was found at 12 months postoperatively, both in plain radiographs and in CT images. New bone was greater at the opposite side of the plate.

**Figure 6:**
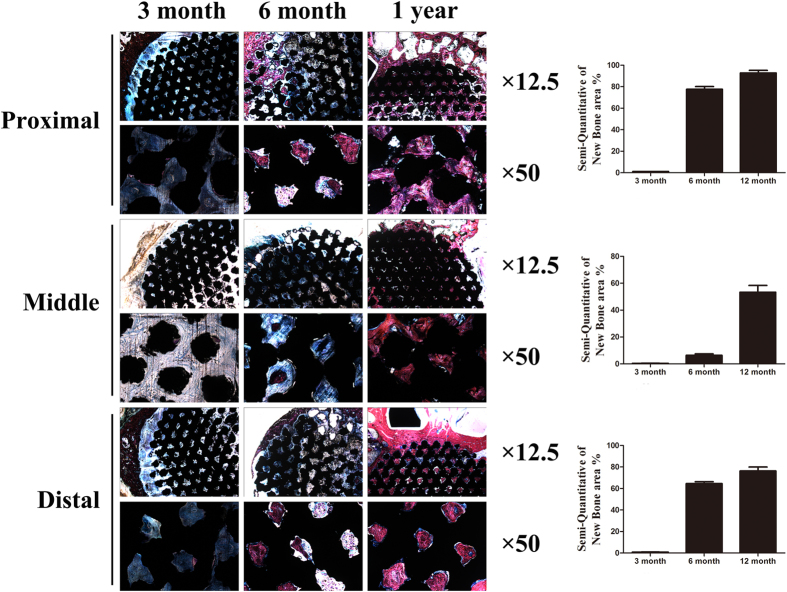
Histologic sections of porous Ti6Al4V scaffolds implanted into goat metatarsus large segmental defects and semi-quantitative of new bone area in the scaffolds. At 3 months, callus in the periphery regions had formed; apparent bone ingrowth was observed at 6 months; at 12 months, the inner space of the scaffolds was nearly completely filled with bone tissue. New bone area increased with time and, compared with the middle position, both ends had relatively more amounts of new bone, with the proximal position being superior to the distal position. Stain: Stevenel’s blue and Van Gieson’s picrofuchsin. Purple indicates bone; black indicates materials; blue indicates fibrovascular tissue.

**Figure 7:**
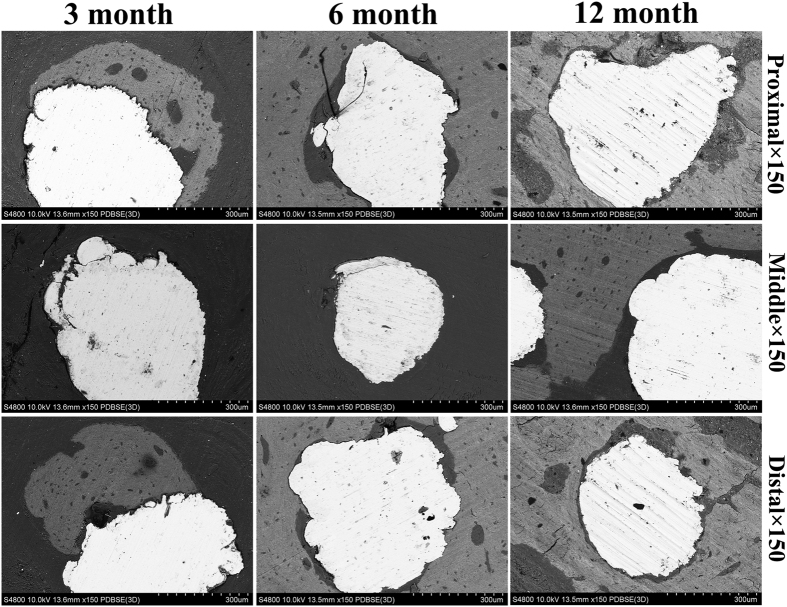
SEM micrographs of bone apposition and bone microstructure on porous scaffolds in different positons at 3 month, 6month and 12 month. White indicate Ti6Al4V, grey indicate new bone.

Histological images of each of the time points (3, 6 months and 12 months) are shown in Fig. 6. At 3 months, both ends of the implants were surrounded with bone callus in the peripheric regions, while at the middle position of the implants, no callus was found. The inner porous areas of the implants were filled with fibrovascular tissue, and sporadic new bone could be found clinging to materials at high magnification. At 6 months, new bone had grown into the inner pores at both ends of the implants. However, this new bone was immature woven bone, and bone ingrowth only occurred near the peripheric regions. The inner porous areas were still filled with fibrovascular tissue; however, in some pores, new bone could be seen clearly and higher amount of new formed bone was observed at 6 months than at 3 months. At 12 months, both the ends and the middle position of the implants were filled with new bone, which had remodeled into mature lamellar bone; the same characteristics were found in the inner porous areas. The continuity of bone from proximal to distal had recovered through the porous implant. Bone apposition and bone microstructure could be observed in Fig. 7. At 3 months, bone apposition was only located at both ends of the implants, but with time going, the inner porous areas were also filled with new bone, which was consistent with the histological images. Direct bone-implant contact could be observed in the images at each time points.

Quantitative analysis results of the bone ingrowth area are shown in Fig. 6. In general, the bone ingrowth area was increased over time at all three positions. At each time point, the bone ingrowth area was significantly higher at both end positions than at the middle position. While comparing the two ends, the proximal region was significantly higher than the distal region.

## Discussion

The current experimental results demonstrated that porous Ti6Al4V alloy scaffolds fabricated by EBM can be tailored to have better cytocompatibility and mechanical-adapted properties for repairing large segmental bone defects.

Implants that are generated with a porous structure can decrease stiffness and avoid the stress shielding effect. Interconnected pores are conducive to nutriment and oxygen exchange in the process of cell metabolism. In addition, a porous structure can offer space for bone ingrowth and vascularization, which can lead to biological fixation. Previous research demonstrated that a pore size greater than 100 μm is suitable for cell migration and transport^27^. In addition, the macroporous structure (>200 μm) is important for capillary tissue and osteoprogenitor cell migration into porous spaces^27^. To explore the specific suitable pore size and porosity, here, we designed three different pore sizes (300~400, 400~500, and 500~700 μm), all within the scope mentioned above; with the aid of EBM technology, the mean pore size of porous samples is controlled more accurately to values of 315, 485, 574 μm, respectively. This indicated that EBM was a feasible approach for processing a porous structure with a gradient pore size and porosity, which was also demonstrated by the well-controlled interconnectivity from specimen histological sections.

The premise of bone ingrowth is that cells adhere to the surface of materials and migrate to the inner space, which has an important relationship with the characteristics of the porous structure. The present results indicated that cell adhesion occurred on all three porous samples based on the SEM morphology, and the group of intended pore size of 300 to 400 μm was more favorable for cell adhesion. The results further confirmed that the characteristic of the porous structure had a significant influence on cell adhesion. Cells on porous samples exhibited a well-spread shape and were able to modify their morphology to follow the topography of the space. Under the condition of guaranteeing cell migration to an inner space, a porous structure with more surface area will exhibit higher cell viability. The surface area was inversely proportional to the pore size; in the current study, porous samples in the group of intended pore size of 300 to 400 μm provided a larger surface area for cell adhesion; thus, the present results could be explained and were in accordance with those previous studies^32^,^33^. Furthermore, the group of intended pore size of 300 to 400 μm offered an advantage with respect to curvature. As previously reported, a higher average curvature induced higher tissue amplification *in vitro*^34^,^35^. If the same structure was used with different pore size, the cross sectional shape was the same, while the average curvature of the pore became higher in inverse proportion to pore size^35^,^36^. Considering these factors, the group of intended pore size of 300 to 400 μm was more advantageous in terms of the surface area and the curvature. The present SEM morphology and cell proliferation results of the group of intended pore size of 300 to 400 μm might be mainly explained by the beneficial effect of a higher surface area and higher curvature.

The present results clearly showed that at day 7, the ALP activity of the group of intended pore size of 300 to 400 μm was significantly higher than that of the other two porous groups; this result can be interpreted in several aspects. First, this porous sample had the appropriate pore size, allowing for cell migration to the inner space and simultaneous spanning of the pore at an earlier period. Both at the inner space or the surface area, the scaffold provided a good environment for cell adhesion and proliferation. Second, to adapt to the space topography, the cell morphology was changed, thereby enhancing cell differentiation, as reported previously^37^. Last, cells on this porous sample became confluent according to the SEM morphology, which is a prerequisite for the development of signal transduction in the process of osteogenic differentiation. The results of the semi-quantitative analysis of mineralized nodules at day 21 indicated that there were no significant differences in the three porous groups. As we know, during the process of osteogenic differentiation, both the porous structure and the osteogenic differentiation medium can exert an influence on the final result. However, accurate explanation of this result needed further study.

When fabricating an implant for repairing bone defects, both the cytocompatibility and mechanical properties should be taken into account. The elastic modulus of porous Ti6Al4V alloy samples produced via EBM could be decreased accordingly to match that of natural bone. The elastic modulus of human bone has a certain difference using different detection methods^38^. In general, human cancellous bone has an elastic modulus of less than 3 GPa, and cortical bone has an elastic modulus of less than 20 GPa^8^,^26^. The elastic modulus of all three of the porous samples that we designed was 3.7, 2.3, 1.7 GPa, and the group of intended pore size of 300 to 400 μm was more close to human cortical bone values compared with the other two porous scaffolds; thus, the elastic modulus could be used as an important parameter in fabricating implants for repairing large segmental bone defects in load-bearing areas. Furthermore, the elastic modulus of porous Ti6Al4V scaffold demonstrated in this study was consistent with early report^28^. Previous research studies reported that the elastic modulus of sheep metatarsus cortical tissue was 18.96 GPa according to the tension and bending test; this value was very close to that of human bone^39^. In fact, the use of such porous samples for repairing metatarsus large segmental bone defects in the current study exhibited a promising effect based on the results of X-ray and histological evaluations. The results also verified the validity of the choice of animal models for offering a similar biomechanical environment as that of the human situation.

The mechanical-adapted porous implant acquired good bone ingrowth according to histological evaluations. The tissues that grew in the implant were evenly distributed, which was attributed to the good interconnectivity of implant, demonstrating the advantage of the additive manufacturing method compared to the traditional methods. Bone apposition directly on the implant surface at 3 months postoperatively, and bone callus surrounding the implant was also observed. The depth of bone ingrowth became gradually deeper and the bone area in the implant became larger with time. At each time point, the bone area in the both ends was higher than that in the middle position. which was consistent with the direction of bone growth and remolding. The histomorphometric analysis result indicated that the significantly higher proximal regions compared to the distal regions in terms of the bone area could be mainly explained by the difference of blood supply. Segmental periosteum was damaged when bone defects occurred, and the blood supply of distal region decreased to some extent postoperatively. *In vivo* evaluation of porous Ti6Al4V scaffold prepared by EBM was also conducted in other researches and results demonstrated this scaffold could acquire favorable bone ingrowth after implanted in sheep femora^40^,^41^.

Compared with other synthetic bone graft substitutes, porous metallic scaffolds not only can provide sufficient initial mechanical stability but can also reduce the risks of implant failure due to degradation and can acquire good bone ingrowth, which is conducive to fulfilling biological fixation. In addition, histological evaluations and mechanical investigations are necessary to assess the bond strength at the interface of the implant and the bone, which is connected to the implant stability. And in animal experiments, if more groups with a gradient pore size are designed, then we would be able to investigate the influence of the pore size on osseointegration in the *in vivo* circumstance under load-bearing, which would provide important guidance for clinical implant design. The limitations mentioned above will be explored in a future study, and the details will be presented.

## Conclusions

In summary, we have shown that porous Ti6Al4V scaffolds with various morphology and mechanical-adapted property can be fabricated via EBM. In addition, a scaffold with intended pore size of 300 to 400 μm was considered to be more suitable for the adhesion and proliferation of hBMSCs. Moreover, due to its biocompatibility and mechanical-adapted properties, a 30-mm segmental goat metatarsus bone defect treated with the scaffold was identified as achieve favorable bone ingrowth and implant stability. To our knowledge, there were few studies to show the osteointegration of additive manufactured porous Ti6Al4V scaffold on a loaded bone defect model. Investigated scaffolds can be designed with matched morphology and mechanical-adapted property to provide early load-bearing for large segmental bone defects, while acquiring favorable bone ingrowth. These data support the potential use of additive manufactured porous Ti6Al4V scaffold as bone defects repairing devices.

## Additional Information

**How to cite this article**: Li, G. *et al.* *In vitro* and *in vivo* study of additive manufactured porous Ti6Al4V scaffolds for repairing bone defects. *Sci. Rep.* **6**, 34072; doi: 10.1038/srep34072 (2016).

## Figures and Tables

**Table 1:** Mean pore size, porosity and mechanical properties of porous Ti6Al4V scaffolds.

Group		300–400 μm	400–500 μm	500–700 μm
Pore Size/μm	designed	300–400	400–500	500–700
	manufactured	315 ± 76	485 ± 83	574 ± 49
Porosity/%	designed	80	80	80
	manufactured	33.8 ± 0.8	50.9 ± 0.6	61.3 ± 0.4
Elastic Modulus/GPa		3.7 ± 0.2	2.3 ± 0.1	1.7 ± 0.2
Compressive Strength/MPa		115.2 ± 12.8	51.5 ± 6.4	33.1 ± 5.4
